# Solitary Esophageal Ectopic Sebaceous Glands

**DOI:** 10.31662/jmaj.2025-0222

**Published:** 2025-08-22

**Authors:** Akihiro Okano

**Affiliations:** 1 Department of Preventive Medicine, Sankei Clinic, Kitakatsuragi, Japan; 2 Department of Gastroenterology, Tenri Hospital, Tenri, Japan

**Keywords:** ectopic sebaceous glands, esophagus, solitary, xanthoma

A 67-year-old woman with abdominal discomfort consulted our hospital. Esophagogastroduodenoscopy revealed a rounded, rosette-like, yellowish, elevated lesion, 4 mm in diameter, in the upper esophagus (Figure 1). Histopathologic examination of the biopsy specimen showed lobules of ectopic sebaceous glands (ESGs) within the squamous epithelium (Figure 2). ESGs have been identified in various ectodermal tissues, including the lips, salivary glands, and prepuce. Although ESGs have been reported in the esophagus, their presence remains uncommon because the esophagus is of endodermal origin. There is ongoing debate regarding whether esophageal ESGs result from congenital misplacement or metaplastic change. Reported cases typically involve multiple ESGs ^(1)^, often ranging from 25 to 100 lesions in number. In contrast, solitary esophageal ESGs are particularly uncommon. Generally, esophageal xanthomas present as solitary, granular, white-to-yellowish lesions. Therefore, distinguishing solitary esophageal ESGs from xanthomas before endoscopic biopsy confirmation can be challenging.

**Figure 1.**
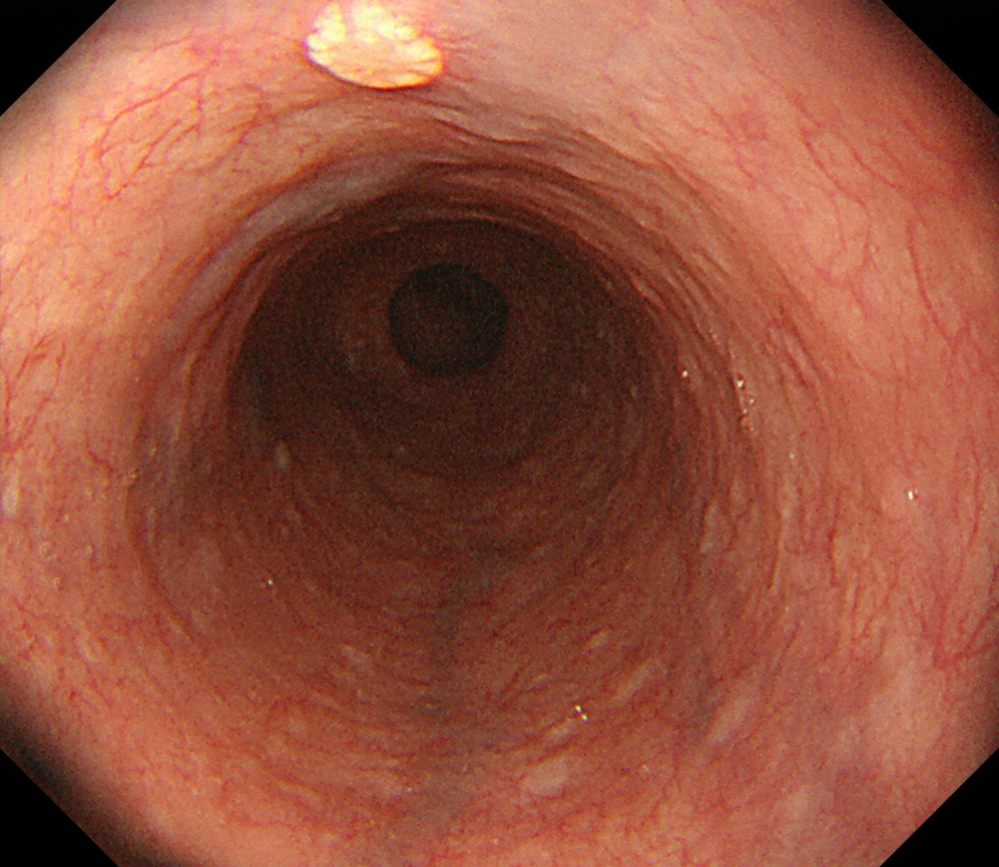
Esophagogastroduodenoscopy revealed a 4 mm, flower-like, slightly elevated, yellowish lesion in the esophagus.

**Figure 2.**
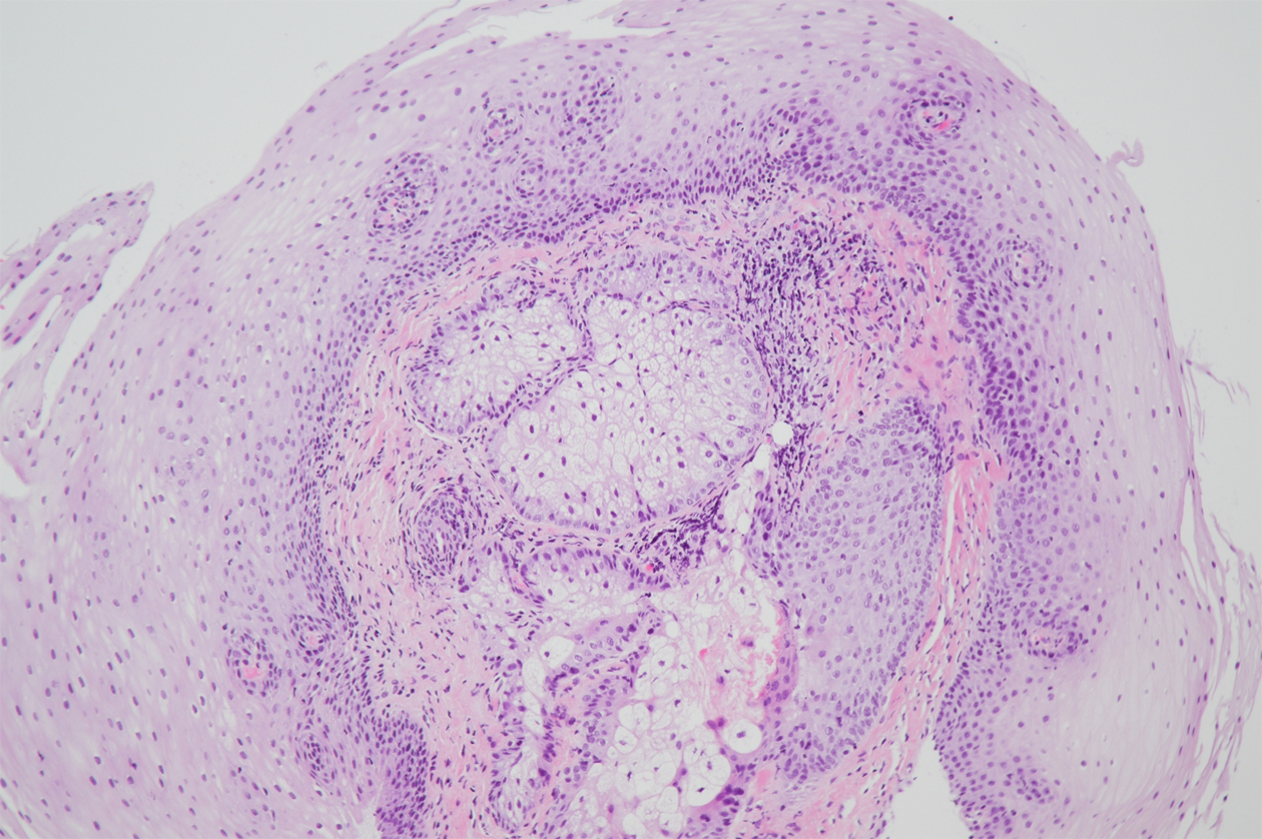
Histopathologic examination showed the ectopic sebaceous gland consisting of lobules of polygonal cells with small round nuclei and abundant clear cytoplasm, covered by squamous epithelium.

## Article Information

### Conflicts of Interest

None

### Author Contributions

Akihiro Okano took charge of all the work.

### Patient Consent

Informed consent was obtained from the patient.
